# Infertility services in the context of decreasing total fertility rates

**DOI:** 10.2471/BLT.25.294210

**Published:** 2025-11-12

**Authors:** Gitau Mburu, James Kiarie, Pascale Allotey

**Affiliations:** a Human Reproduction Programme, World Health Organization, Avenue Appia 20, 1211 Geneva 27, Switzerland.; b Department of Sexual, Reproductive, Maternal, Child and Adolescent Health and Ageing, World Health Organization, Geneva, Switzerland.

The world is undergoing profound demographic transitions, marked by notable changes in fertility patterns, life expectancy and population structures.^1^ Nearly all countries are experiencing a decline in total fertility rate, defined as the average number of children a woman would have if current age-specific birth rates remain constant throughout her childbearing years.^1^ The global total fertility rate is 2.3 births per woman, a rate that is declining by approximately 1% annually.^1^ These changes in total fertility rate are accompanied by improved survival and mortality rates, population ageing and delayed childbearing.^1^^,^^2^

Infertility is an important global health issue that features in the discussions about the contemporary decline in total fertility rates. However, a decline in total fertility rates does not necessarily mean a rise in infertility, as these two phenomena are different. Declines in total fertility rates predominantly occur when individuals give birth to fewer or no children, or have them later in life by either delaying or forgoing parenthood due to structural, social or personal circumstances.^3^ On the other hand, infertility, which affects an estimated 17% of adults during their lifetime,^4^ is mainly a biological issue affecting male or female reproductive functions, characterized by the failure to achieve a pregnancy after 12 months or more of regular unprotected sexual intercourse. 

Contemporary social phenomena such as the postponement of childbearing play a role in both infertility and declining total fertility rates. Postponement of childbearing results in a smaller available window for achieving reproductive goals and a higher likelihood of having fewer children, irrespective of whether the original delay was voluntary or due to circumstantial constraints. Simultaneously, postponement of childbearing has clear biological implications and directly increases the risk of infertility. Female fecundity declines with age due to reduced number and quality of oocytes and other age-related physiological factors. At age 30 years, 75% of women can conceive and give birth within a year, but this figure drops to 66% by age 35 and to 44% by age 40.^5^

These demographic and epidemiological phenomena present both complex challenges and opportunities for health systems. These phenomena demand expanded services for fertility preservation and infertility treatment, but also require safeguarding the principles of rights and reproductive choice, particularly in contexts where governments are increasingly concerned about population dynamics. 

To adapt to fertility constraints, increasing numbers of people are turning to medically assisted reproduction, including intrauterine insemination and a range of other assisted reproductive technologies such as in vitro fertilization, which can partially offset age-related fertility decline. Medically assisted reproduction counteracts age-related effects on fecundity by increasing the number of mature oocytes. The use of in vitro fertilization is rising among women older than 40 years, as more people seek biological parenthood later in life. This microlevel demand has also been accompanied by macrolevel policy interest. Governments are increasingly concerned about fertility, population and other demographic trends, whether high fertility in some regions, declining birth rates in others or the unmet needs related to infertility treatment. In countries facing low total fertility rates, policy-makers are exploring whether medically assisted reproduction might boost birth rates and population sizes. The central question, therefore, is the extent to which medically assisted reproduction influences total fertility rates.

Children born through assisted reproductive technologies comprise a growing proportion of total births in many countries. At least 10 million children have been born globally through these technologies since their advent in 1978, with around 800 000 more born annually.^6^ In high-income settings, assisted reproductive technologies contribute more than 5% of national live births in several countries, with reported contributions ranging from negligible to more than 9%, and most contributions averaging between 2–5% of total live births.^7^^,^^8^

Modelled estimates of assisted reproductive technologies’ contribution to national total fertility rates suggest a modest, though non-trivial effect. In countries where these technologies are relatively accessible, such as Australia, Denmark, Kingdom of the Netherlands, United Kingdom of Great Britain and Northern Ireland and United States of America, use of these technologies enhances the national total fertility rate by 0.02–0.10 children per woman.^8^^–^^11^ While this contribution appears modest at a global scale, it may be statistically and socially significant in countries with very low total fertility rates.

Despite growing interest in the potential of medically assisted reproduction to mitigate declining fertility rates, its overall contribution to total fertility rates remains modest. This limited impact is shaped by several interrelated factors that intersect biological, social, economic and methodological dimensions.

A primary constraint is the inequitable access to fertility care worldwide. While medically assisted reproduction technologies have advanced considerably over the last several decades, utilization remains inequitable, being lowest in the World Health Organization (WHO) African Region and highest in high-income settings.^6^ High costs, inadequate insurance coverage and low availability of medically assisted reproduction, particularly in low-income settings, hinder its use. Without deliberate efforts to improve both its affordability and availability, the reach of medically assisted reproduction will remain confined to a relatively privileged subset of the population.

Biological realities also impose limits on the effectiveness of medically assisted reproduction, particularly with advancing maternal age, since its efficacy reduces with age, even when such technologies are available and accessible. For instance, following two completed in vitro fertilization cycles, the probability of achieving a live birth is approximately 30% at age 34 years, but 24% at age 38, and just 17% at age 42.^5^

Another challenge lies in the widespread over-expectation of medically assisted reproduction success rates. Many individuals delay parenthood under the assumption that technologies such as in vitro fertilization can adequately compensate for age-related declines in fertility. This optimism can contribute to decisions that reduce the likelihood of successful outcomes later in life.

Furthermore, medically assisted reproduction is not universally relevant because fertility intentions differ. For individuals who voluntarily choose not to have children, these services are neither needed nor desired. Total fertility rates reflect the number of children born per woman and do not differentiate between voluntary and involuntary childlessness. Reproductive health policies must therefore be sensitive to the diversity of reproductive intentions, including the choice to remain child-free.

Policy, regulatory and ethical considerations further shape access to and outcomes of medically assisted reproduction. Many jurisdictions impose upper age limits for providing it, in part to protect maternal and neonatal health and optimize its cost–effectiveness. In parallel, safer clinical practices such as single embryo transfer, while crucial in reducing the risk of multiple births and associated complications, can potentially limit the number of live births per treatment cycle. These measures, while necessary, may be perceived as constraining the potential contribution of medically assisted reproduction to total fertility rates, especially in the absence of cumulative live birth rate data.

Finally, significant methodological limitations exist in current efforts to quantify the impact of medically assisted reproduction on total fertility rates. Much of the available data stem from modelling exercises, due to the difficulty of assessing the impact of complex interventions on population outcomes. However, modelling studies rely on assumptions that are often difficult to validate. These models must contend with uncertainties around miscarriage rates, the effects of multiple births, donor gametes, paternal age, behavioural changes, cumulative effects of births, counterfactual births in the absence of medically assisted reproduction, evolving clinical practices and other covariates.^5^^,^^8^^–^^11^ Existing evidence, though informative, remains incomplete and is geographically limited to high-income settings, and may therefore over- or underestimate the utilization of medically assisted reproduction and its subsequent impacts. As more empirical evidence becomes available, better estimates of the impact of medically assisted reproduction on total fertility rates will be possible.

Given that demographic transitions, particularly delayed parenthood and declines in total fertility rates, are likely to persist, health systems ought to adapt proactively, from micro- to macrolevels. Doing so entails expanding local availability, eligibility, diagnostics and insurance coverage related to infertility to ensure that those who need diagnosis and treatment can afford and universally access it. Shifting responses from treatment of infertility to preventing it from occurring is also essential. WHO’s infertility guidelines recommend preventing infertility through timely treatment of sexually transmitted infections, supporting lifestyle adjustments (in diet, alcohol intake, smoking, physical activity and/or weight management) and providing adequate information on fertility and infertility, while embedding fertility care within a broader sexual and reproductive health strategy that respects individual rights and promotes informed choice.^12^ Low-cost strategies to educate populations of reproductive age about fertility and infertility (and subsequent referrals as needed) can include providing information in digital application or paper format when opportunities occur in educational institutions, at primary health-care centres or at reproductive health clinics.^12^ Fertility education should be made universally accessible, enabling individuals to understand age-related reproductive risks, the realities and limitations of medically assisted reproduction and the full range of available options. Crucially, responses to declining fertility must not devolve into coercive pro-natal policies that facilitate childbirths while restricting the reproductive choices of individuals. The goal of reproductive health services should remain the fulfilment of individual fertility preferences, whether that means having children, delaying parenthood or choosing not to have children at all.

Health system interventions alone cannot reverse declines in total fertility rates. While medically assisted reproduction is an essential component of reproductive health care, adaptation to fertility transitions requires broader, multisectoral efforts. These include enabling environments for parenthood, such as through affordable childcare, paid parental leave, workplace flexibility, housing support, and inclusive and gender-equitable family policies that reduce the structural disincentives to having children, among other interventions.

In conclusion, medically assisted reproduction plays an important, albeit modest, role in offsetting declines in overall fertility rates. Although its contribution to total fertility rates is modestly growing, it remains constrained by biological, financial, structural, social and behavioural factors. Nevertheless, medically assisted reproduction is a critical part of comprehensive reproductive health care and must be integrated into national health systems as a rights-based, accessible and quality-assured service. Medically assisted reproduction should be viewed as part of holistic fertility care that includes prevention, diagnosis and treatment interventions. In turn, fertility care, health systems and wider multisectoral interventions are interlinked within a compendium of adaptive policy options, none of which, alone, constitutes an effective response to demographic shifts. Declines in total fertility rates need to be met with diverse policies and interventions that enable informed, voluntary and supported reproductive decision-making for all.
